# SL-scan identifies synthetic lethal interactions in cancer using metabolic networks

**DOI:** 10.1038/s41598-023-42992-4

**Published:** 2023-09-22

**Authors:** Ehsan Zangene, Sayed-Amir Marashi, Hesam Montazeri

**Affiliations:** 1 Department of Bioinformatics, Institute of Biochemistry and Biophysics, University of Tehran, Tehran, Iran; 2 Department of Biotechnology, College of Science, University of Tehran, Tehran, Iran

**Keywords:** Biochemical reaction networks, Cancer, Cancer genetics, Cancer genomics, Computational models, Systems biology

## Abstract

Exploiting synthetic lethality is a promising strategy for developing targeted cancer therapies. However, identifying clinically significant synthetic lethal (SL) interactions among a large number of gene combinations is a challenging computational task. In this study, we developed the SL-scan pipeline based on metabolic network modeling to discover SL interaction. The SL-scan pipeline identifies the association between simulated Flux Balance Analysis knockout scores and mutation data across cancer cell lines and predicts putative SL interactions. We assessed the concordance of the SL pairs predicted by SL-scan with those of obtained from analysis of the CRISPR, shRNA, and PRISM datasets. Our results demonstrate that the SL-scan pipeline outperformed existing SL prediction approaches based on metabolic networks in identifying SL pairs in various cancers. This study emphasizes the importance of integrating multiple data sources, particularly mutation data, when identifying SL pairs for targeted cancer therapies. The findings of this study may lead to the development of novel targeted cancer therapies.

## Introduction

Identifying cancer dependencies through analysis of large-scale perturbation studies is an active research field with the potential to discover new therapeutic targets for cancerous tumors ^1,2^. Several large-scale shRNA and CRISPR-Cas9 perturbation screenings have been conducted to identify novel cancer genes and dependencies ^3,4^. Through the analysis of these screens, researchers have made significant progress in unraveling the complex biology of cancer and identifying novel therapeutic targets ^1^. To analyze diverse experimental data such as gene expression, mutation, and drug response, a range of statistical and machine learning models have been developed, including models to identify associations between cell line viability and molecular features and models that predict drug responses based on genomic features ^5–7^. Moreover, several computational tools have been proposed for uncovering SL interactions, which are critical for discovering personalized cancer therapies that exploit cancer-specific vulnerabilities. Synthetic lethality refers to a genetic interaction between two or more genes where the loss of function, either through genetic alterations or inhibition, in all genes is required to cause cell death^8^. This concept provides a novel approach to combating cancer, but only a handful of inhibitors that target partner genes in SL interaction, such as BRCA1/2-PARP1, have received FDA approval for use as SL-based therapies^9–11^. Identifying clinically significant SL interactions is a challenging task through experimental validation, due to their rarity, as well as the high cost and time requirements associated with wet lab experiments ^12,13^. Therefore, various computational approaches have been developed to address this challenge and facilitate the identification of clinically relevant SL interactions in cancer cells. SLIdR is a rank-based statistical method that predicts SL pairs from shRNA perturbation screens in both pan-cancer and cancer-specific settings ^2^. MiSL is another computational tool that identifies SL partners for specific cancer mutations in particular cancer types using pan-cancer human primary tumors ^14^.

Additionally, several approaches have incorporated genome-scale metabolic networks (GSMN) to investigate the effects of gene knockdown on cancer cell line growth rates and to predict cancer vulnerabilities and potentially identify novel targeted therapies^15–17^. In addition, cancer genome-scale metabolic modeling has been employed to predict SL pairs in various cancer types^9,18,19^. The process of cancer constraint-based metabolic modeling involves tailoring a cancer-specific metabolic model from a generic model and omics data. This tailored model can then be subjected to various analyses based on objective functions such as biomass production, which is a well-established objective in cancer metabolic modeling due to its close association with cell growth rate^20,21^.

There are two metabolic network approaches for predicting SL pairs in different types of cancer: the minimal cut set (MCS) and exhaustive search algorithms. The MCS algorithm identifies sets of reactions that, if removed, would prevent the execution of a specific metabolic function, such as the biomass reaction. Klamt et al. developed the MCS algorithm for SL identification using the enumeration of shortest elementary modes in a dual representation of metabolic networks^22^. In contrast, the gene minimal cut set (gMCS) algorithm searches for MCS in gene level ^18^. The ngMCS algorithm, an extension of gMCS, incorporates environmental factors in the SL prediction. By considering both gene function loss and nutrient absence, ngMCS incorporate both genetics and the microenvironment for predicting metabolic SL interactions ^23^. On the other hand, the exhaustive search algorithm aims to identify pairs of reactions whose deletion significantly reduces or nullifies the objective function compared to the wild-type model^24,25^. Fast-SL is an exhaustive search strategy that narrows the solution space to find SL pairs efficiently^26^.

However, these methods exhibit certain limitations. Firstly, these approaches primarily lean on metabolic models contextualized with expression data, often overlooking the inclusion of mutation data. Integrating mutation data could potentially yield improved SL predictions across different cancer types. Secondly, metabolic network-based SL prediction algorithms typically present SL pairs in a deterministic manner, whereas the incorporation of a concept of statistical significance would be more desirable for addressing data uncertainty and imprecision of methodological approaches.

This paper presents the development of SL-scan, a computational pipeline that integrates metabolic network modeling and mutation data to identify SL pairs in various cancer types. The approach involves tailoring a generic human metabolic model using iMAT to construct a cancer-specific metabolic model for each cancer cell line. Gene knockouts are then simulated for all genes, and simulated dependency scores are estimated based on the constructed cancer metabolic models for each cell line separately. We then predicted potential SL interaction by evaluating the relationship between the mutation statuses of a driver gene across different cell lines and the simulated dependency scores of a potential SL partner gene. Our results demonstrated that SL-scan outperformed existing SL prediction algorithms in metabolic modeling and successfully identified putative SL pairs in various cancer types, as confirmed by benchmarking against CRISPR, and drug perturbation data analysis. These findings provide valuable insights into the discovery of novel metabolic-based cancer therapies.

## Material and methods

### Datasets

**Figure 1:**
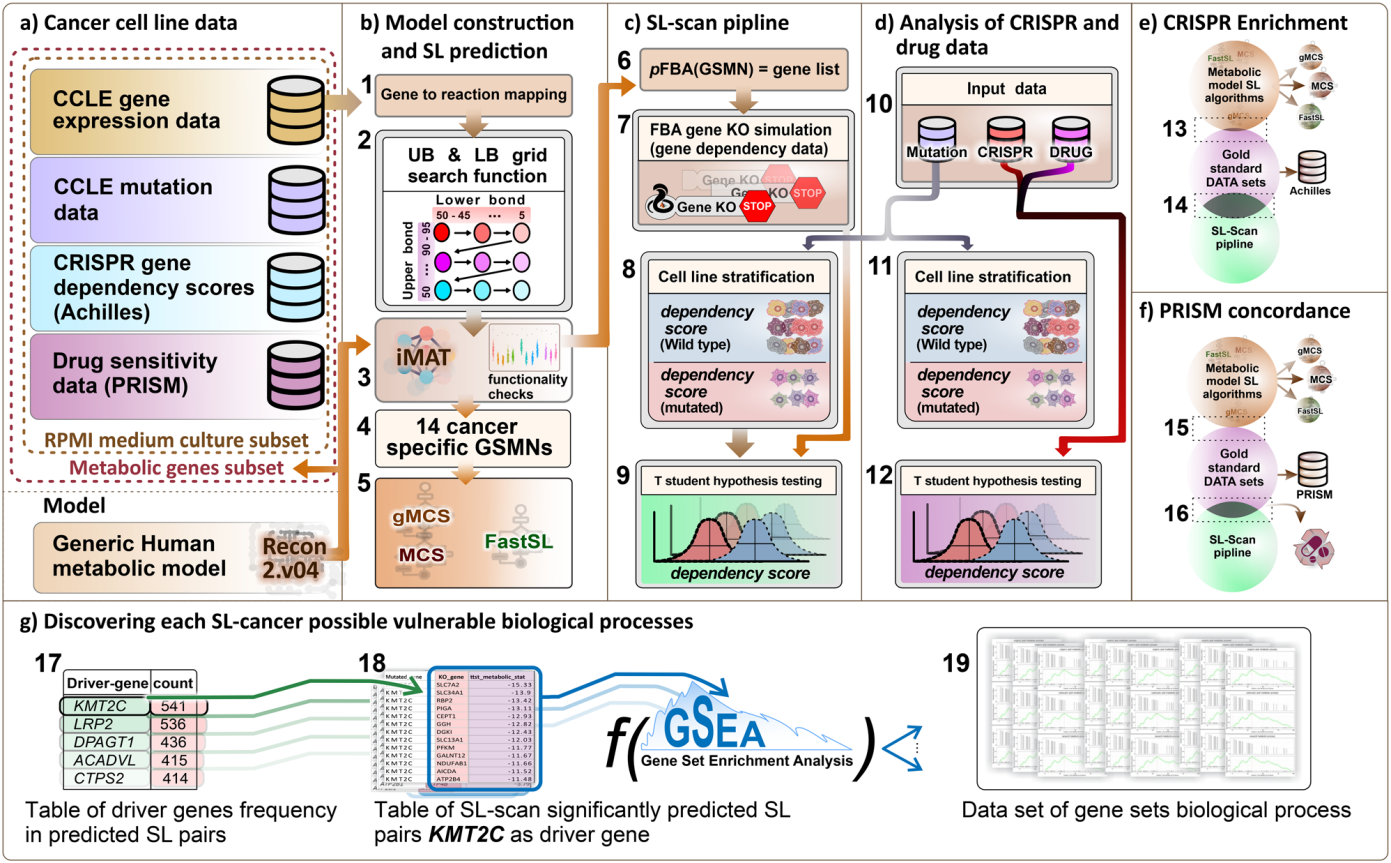
Illustrations of the different stages of the study. (**a**) The input datasets were filtered to only include metabolic genes and cancer cell lines cultured in RPMI with at least 10 samples. (**b**) The MCS, gMCS, and FastSL SL prediction algorithms were used to predict SL pairs for 14 cancer types that were constructed using iMAT. (**c**) Gene dependency matrices were simulated using metabolic models. Using the mutation dataset, cell lines were stratified based on whether they have mutated or wild-type driver genes, and a t-test was performed for the gene pair. d) Identification of SL interactions with PRISM drug perturbation sensitivity and CRISPR cancer gene dependency matrices. e) Assessment of overlaps of predicted SL pairs by SL-scan and other algorithms with SL interactions found by analyzing the CRISPR data. f) The same analysis as panel e, but on the PRISM data. g) Gene set enrichment analysis on SL partner genes for each driver gene.

The gene expression data, mutation data, CRISPR, and drug perturbation data sets used in this study were obtained from the Depmap project https://depmap.org/portal/download/all/. The gene expression data set consists of the log2 transformed transcript per million (TPM) values of 19,221 protein-coding genes from 1406 cell lines across 33 cancer types. We obtained the mutation data from the MAF file CCLE_mutations_21Q4.csv, which contains information on all somatic point mutations called in the DepMap cell lines ^27^. We used Achilles_gene_effect.csv (22Q2) for the CRISPR data set, which includes the effects of CRISPR screening on 18,018 genes in 957 cancer cell lines^3^. The drug perturbation data used in this study is the primary PRISM Repurposing dataset, which contains the results of pooled-cell line treatment with chemical perturbation for 4,686 compounds against 578 cell lines. The values reported in this dataset represent the response of each compound-cell line combination, indicating how the compound influenced cell viability. This data reflects the growth inhibitory effects of various compounds on cancer cell lines, measured in terms of log-fold changes in cell viability compared to DMSO-treated cells ^28^. For the integration of shRNA-related information, we incorporated the DRIVE_ATATRiS_data.rds (version 5, Published on 24 Jul 2019) dataset. This dataset encompasses cancer dependency scores originating from the effects of shRNA-induced silencing on 7,837 genes across 398 distinct cancer cell lines. For generating cancer-tailored models, the generic input metabolic model Recon2.v04, obtained from https://www.vmh.life/files/reconstructions/Recon/2.04, was used ^29^. Recon2.v04 includes a total of 2140 genes, 5063 metabolites, and 7740 reactions. For our analysis, we specifically focused on the metabolic genes contained in the Recon2.v04 gene list. As a result, the overlap of mutation data, CRISPR dataset, and the metabolic network gene list resulted in 1674 shared genes. Likewise, the intersection of the expression dataset, CRISPR dataset, and mutation data comprised 850 unique cancer cell lines. To ensure that the culture medium does not impact the predictive potential of the SL algorithm, we restricted our analysis to cancer cell lines that were cultured in the RPMI culture medium. Additionally, we only included cancer cell lines that had at least ten observations in the gene expression data. To implement this, we followed the step-by-step procedure outlined in the article by Jamialahmadi et al.^30^. We used the MutSig files to define significantly mutated genes in TCGA for individual cancer types. Supplementary Table S1 relates the cancer types as defined within the DepMap project and their corresponding counterparts in the TCGA datasets (Fig. 1a).

### Flux balance analysis

Flux Balance Analysis (FBA) is a linear programming technique extensively employed in analyses of metabolic network. FBA facilicates understanding of cellular metabolism by optimizing the flow of metabolites through reactions within metabolic networks. The constrained optimization problem of FBA is given as follows:

1 $$ \begin{gathered}   {\text{maximize}}~~c^{T} x \hfill \\   {\text{subject}}\;{\text{ to}} \hfill \\   Sx = 0 \hfill \\   l \le x \le u \hfill \\  \end{gathered}   $$

In this formulation, 'c' denotes a vector of coefficients that indicates the respective contributions of individual reactions to the objective function, x is the reaction flux vector, S is the stoichiometric matrix, and l and u are lower bounds and upper bounds for reaction fluxes, respectively. The solution x captures the optimized reaction fluxes under the above constraints.

The stoichiometric matrix (*S*) is a key component in FBA, representing the stoichiometry coefficients of each metabolite participating in the network reactions. Each row of the matrix corresponds to a metabolite, and each column corresponds to a reaction. The elements of *S* indicate the stoichiometric coefficients of each metabolite in a given reaction. The stoichiometric matrix forms the basis for mapping the connectivity and flow of metabolites in the network.

The Gene-Protein-Reaction Association (GPRA) rules specify a connection between genes, proteins, and reactions within a metabolic model. These rules define which genes encode enzymes responsible for catalyzing specific reactions. GPRA rules enable the translation of genetic information into the context of metabolic reactions, thereby facilitating the integration of gene expression data into metabolic models^31–33^.

### Cancer model construction

We used gene expression data to tailor the Recon2.v04 human metabolic model and create cancer-specific metabolic models using the iMAT algorithm, which has been shown to maintain more cancer-specific genes^34^. The iMAT algorithm used both Upper Bound (UB) and Lower Bound (LB) reaction expression thresholds. For each cancer type, we conducted a grid search to identify the first suitable UB and LB thresholds. These thresholds were determined based on their ability to successfully solve an FBA simulation with a non-zero objective function value. This iterative process allowed us to optimize the UB and LB values for each specific cancer type, ensuring accurate modeling of cellular metabolism and achieving meaningful simulation results. The createTissueSpecificModel function in the COBRA toolbox was used to construct the cancer-specific models, taking into account the UB and LB thresholds, a reaction expression vector, the generic model, and a core set of reactions as inputs. This approach has proven to be useful in cancer metabolic modeling (Fig. 1b) ^30,33,35^.

### Metabolic models evaluation

We verified the structural soundness of all constructed models for both linear programming and quadratic programming problems using the verifyCobraProblem function. Additionally, we used the verifyModel function, a built-in function of the COBRA toolbox, to ensure the reaction flux consistency, mass balance, and the presence of necessary fields in the resulting models. Using Opdam et al.'s definition of the 56 metabolic-based functionalities essential for cancer cell proliferation, we evaluated each constructed model. We compared the number of functionalities of each model against 100 random models with the same size. A cancer model construction was considered successful if its number of passed functionalities is larger than 99% of the numbers of functionalities passed by random models^36^.

### MCS, gMCS, ngMCS, and FastSL

We employed MCS, gMCS, ngMCS, and FastSL algorithms for the SL identification. We implemented MCS and gMCS using the COBRA-toolbox's built-in functions. We followed the guidelines outlined on the relevant GitHub page to use FastSL, which can be accessed at https://github.com/RamanLab/FastSL. The FastSL method uses the default cutoff value of 0.01. The ngMCS related functions were implemented using supplementary files of the corresponding article^23^. All the SL prediction algorithms were restricted to searching for cut sets or SLs with a maximum of two genes. Moreover, a time limit of four minutes is designated for solving the optimization problems of gMCS, MCS, and ngMCS algorithms. These algorithms were configured to predict a maximum of 1000 SL pairs. For translating reaction-level SL pairs to gene-level pairs for the FastSL and MCS algorithms, we used the 'findGenesFromRxns()' function of the COBRA toolbox. This function applies the GPRAs of the Recon2.v04 metabolic model. For the ngMCS algorithm, which predicts metabolites involved in SL pairs, we initially identified the reactions using the predicted metabolites with the 'findRxnsFromMets()' function of the COBRA toolbox. Subsequently, we then converted these reactions into genes using the 'findGenesFromRxns()' function(Fig. 1b).

### SL-scan pipeline

The SL-scan has the following steps:

- *Step 1:* We converted each cancer cell line's gene expression into a metabolic model. To minimize the Taxicab norm of reactions in the FBA problem of a constructed model using the COBRA Toolbox, we used the optimizeCbModel function with the minNorm parameter set to 'one', which solves an LP problem that minimizes the sum of absolute values of fluxes, subject to the constraints of the FBA problem. Mathematically, the optimization problem is defined as:
  2 $$ minimize \,\,  \Sigma \left| v \right| $$
  $${\text{subject to}}$$
  $${v}_{bio} = {v}_{bio,WT}$$
  $$Sx=0$$
  $$l \le x\le u$$
  where ${v}_{bio,WT}$ is the solution for the optimization problem defined in Eq. (1). This equation enables the minimization of the L1 norm of the flux vector (v) to effectively reduce the number of non-zero elements of v. We then transformed the resulting reaction set into a gene set known as the Knockout gene (KO gene) list, using the GPRA rules (Fig. 1c).
- *Step 2:* We conducted simulations to assess the growth rate of the metabolic models both before and after each gene knockout from the KO-gene list. This process generated a KO-simulated score matrix. For genes not included in the KO-gene list but present in the generic model, we assumed that they had no inhibitory effect on growth. Therefore, we assigned a value of 1 to those genes, indicating that their knockout had no impact on the model's growth rate compared to the wild type (Fig. 1c).
- *Step 3:* We then used the mutation dataset to stratify cancer cell lines into mutated and wild groups for each driver gene. We define driver genes as metabolic genes that harbor at least one damaging mutation in at least two cell lines for specific cancer types.
- *Step 4:* To compare the KO-simulated scores between the wild-type and mutated groups, we conducted a t-test for each pair of KO and driver genes. To enhance the efficiency of the SL-scan pipeline in the exhaustive search, we implemented a filtering to exclude driver genes that had a mean greater than 0.95 in the mutated group. In these cases, we assumed a P-value of one. Furthermore, we introduced uniform noise ranging from 1e-12 to 1e-11 to both the wild-type and mutated groups. This adjustment was made to accommodate the metabolic network's tendency to generate KO ratio values with a standard deviation of zero in a small fraction of cases. Furthermore, we used the Benjamini and Hochberg approach to address multiple testing issues and control for false discovery error rate (Fig. 1c).

### Analysis of the CRISPR dataset

To assess the accuracy of the GSMN-based SL pair predictions, we constructed a gold standard using CRISPR perturbation data. We divided the cell lines into wild-type and mutated groups for each driver gene and conducted a t-test on the CRISPR gene dependency scores between the two groups of cell lines for each KO gene. Next, we employed a hypergeometric test to determine whether the CRISPR predicted SL interactions were enriched in the list of SL-scan SL interactions (Fig. 1d-e).

### Analysis of the PRISM dataset

We validated the predictions of GSMN-based SL pairs by cross-checking with the PRISM dataset. We classified cell lines according to the mutation status of driver genes and subsequently conducted t-tests on the PRISM log fold-change values of perturbed genes. We evaluated the enrichment of PRISM-predicted SL interactions among SL-scan predictions using hypergeometric tests (Fig. 1d and f).

### Analysis of the shRNA dataset

We employed ATARiS cancer dependency scores to evaluate the influence of gene silencing through shRNA reagents on cancer cell lines^37^. Similar to our analysis on the CRISPR dataset, we employed mutation data from cancer cell lines to identify driver genes for each cell line. Subsequently, a t-test was employed to assess the influence of gene silencing in cancer cells harboring driver genes with damaging mutations compared to those without such mutations.

### Analysis of target and driver genes in the SL-scan predicted Sl pairs

To thoroughly analyze the involvement and the presence of targets within the context of our predicted SL pairs, we focused on the top 1st quantile of frequently KO genes as determined by the SL-scan pipeline. We provided the associated PubMed IDs to indicate supporting literature for each KO gene. In addition, we also annotated whether a driver gene is a significantly mutated gene in the TCGA dataset using the MutSig files for each cancer type. A gene with a q-value of 0.05 or lower was identified as a significantly mutated gene in TCGA.

### Gene set enrichment

The goal of this step was to discover significant biological processes connected with cancer-driver genes. To this end, a ranked gene list was generated for each driver gene. This involved compiling a list of SL partner genes alongside their corresponding t-statistic values in relation to the driver gene (Fig. 1g). Additionally, we included the driver gene itself in the list, assuming its corresponding score to be the average of t-statistic values obtained when testing with SL partner genes. We sorted the list descendingly according to absolute t-statistic values. We next used the clusterProfiler package function to perform gene set enrichment analysis to identify significant biological processes for each driver gene. Following that, we calculated the frequency of each biological process across all driver genes to determine which were recurrently enriched for driver genes.

## Results

### FBA simulations, consistency checking, and metabolic functionality tests

**Figure 2:**
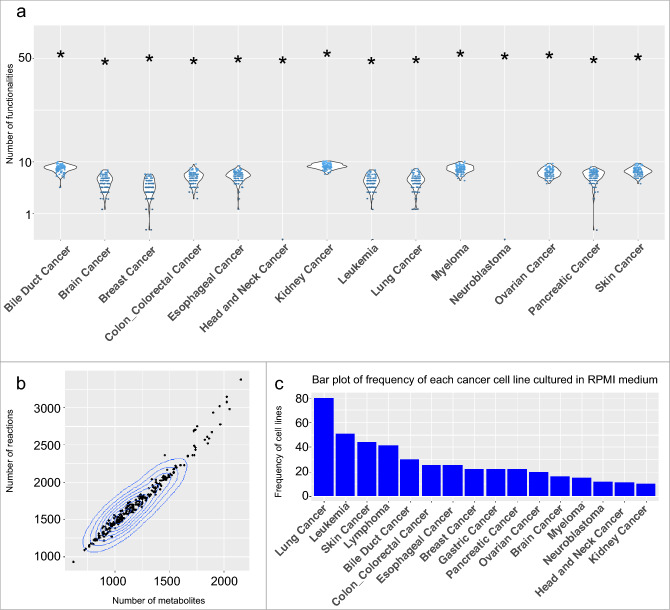
(**a**) Violin plots showing the number of passed functionality tests from random models. The black asterisks indicate the number of passed functionalities in the 14 cancer metabolic models used for gMCS, MCS, and FastSL. (**b**) 2D density plot of the number of reactions and metabolites in the constructed models of the SL-scan pipeline c) The number of cell lines per cancer type used by SL-scan is displayed.

After applying filtration steps to identify relevant cancer types, we have selected a total of 16 distinct types of cancer for inclusion in our study. Due to the inability to provide viable models in the iMAT grid search for UB and LB thresholds, Gastric, and Lymphoma cancers were excluded from the subsequent analyses (see "Materials and Methods" section). We conducted FBA simulations, consistency checking, and metabolic functionality tests to validate each reconstructed metabolic model. We used the COBRA toolbox built-in functions for consistency checking and FBA simulation. Notably, the outcomes of the FBA simulations resulted in non-zero values for all 14 cancer-specific models. In these simulations, we used the default objective function, which was biomass production. Furthermore, the process of consistency checking revealed that all constructed models' reactions remained entirely unblocked, ensuring that fluxes consistently maintained non-zero values. This rigorous validation process reinforces the reliability and robustness of our metabolic models. The fraction of the 56 essential metabolites synthesizing capabilities for each metabolic cancer model is shown in Fig. 2a (See Methods-Metabolic models evaluation). In terms of functionality testing, the constructed cancer-specific models outperformed all of the randomly generated models. The 2D density plot in Fig. 2b displays the distribution of reactions and metabolites in the constructed models for the SL-Scan pipeline. Most models fell within the range of 1000 to 3000 reactions and 1000 to 2000 metabolites. However, one model with 7440 reactions and 5063 metabolites was excluded from the plot to maintain clarity and visual consistency. Additionally, Supplementary Figures S1 and S2 illustrate the distribution of the number of metabolites and reactions of GSMNs across various types of cancers. The number of cell lines per cancer type used by SL-scan is displayed in Fig. 2c.

To identify SL interactions between driver genes and KO genes, our initial step involved constructing a matrix through simulating gene knockout effects using metabolic models. We considered all the metabolic genes within the Recon2.v04 model, resulting in a testing of 1674 KO genes across all cancers. Additionally, the number of driver genes varied for each cancer type, as depicted in Figure S3.

### Validation using CRISPR data

To validate SL predictions with CRISPR data, we used Achilles dependency scores and mutation datasets, along with the Recon 2.v04 model gene list. Table 1 presents the top two SL-scan predicted SL pairs, along with their corresponding adjusted p-values, which align with the CRISPR findings for each cancer type. For a comprehensive collection of results, please refer to supplementary data 1 in the supplementary material. Supplementary Table S2 contains 31,159 significant SL-scan SL pairs, out of which 597 pairs exhibit concordance with the CRISPR outcomes. Notably, the most frequently observed cancers in the context of SL prediction are lung cancer and leukemia, with 181 and 140 concordant SL pairs, respectively. Conversely, kidney cancer and neuroblastoma only display one concordant predicted SL pair each.

**Table 1:** The top two concordant predicted SL pairs by SL-scan and CRISPR analysis for each cancer type.

Cancer	Driver gene	KO gene	CRISPR P-value FDR	SL-scan P-value FDR
Bile Duct Cancer	*PLCG1*	*EPX*	1.64E-07	5.89E-08
	*PLCG1*	*LDHAL6A*	7.68E-07	7.46E-06
Brain Cancer	*ACOX1*	*STRA6*	2.57E-02	2.03E-15
	*AADAC*	*SLC6A20*	2.04E-06	5.54E-15
Breast Cancer	*SLC15A2*	*CYP3A4*	1.61E-09	2.34E-08
	*SLC4A3*	*GSR*	3.78E-03	7.38E-08
Esophageal Cancer	*VNN1*	*LDHD*	4.95E-02	1.12E-04
	*SLCO1C1*	*GUK1*	1.30E-02	2.83E-04
Gastric Cancer	*MAN2B1*	*SLC6A19*	2.25E-03	3.63E-08
	*PIK3C2A*	*ALDH1L2*	2.07E-02	2.90E-06
Head and Neck Cancer	*XDH*	*PRDX3*	1.01E-03	8.61E-11
	*ENTPD2*	*AGXT*	5.63E-03	1.52E-07
Kidney Cancer	*ENGASE*	*PGAP1*	6.43E-03	2.92E-07
	*DPAGT1*	*CNDP1*	1.46E-09	4.76E-06
Leukemia	*PI4KA*	*ALDH3B2*	1.68E-05	1.24E-16
	*PLCE1*	*ALAS2*	5.95E-03	7.72E-16
Lung Cancer	*SLC29A2*	*ISYNA1*	6.11E-08	2.45E-75
	*NT5C1A*	*GMPPA*	1.06E-04	1.25E-36
Lymphoma	*XYLT2*	*TYRP1*	8.31E-03	1.04E-07
	*UGT2B28*	*PLCB4*	4.83E-02	2.18E-06
Neuroblastoma	*LRP2*	*KYAT3*	4.05E-02	5.50E-03
	*ACACA*	*CHPT1*	3.18E-05	9.79E-03
Ovarian Cancer	*SLC16A7*	*PIK3C2G*	1.93E-03	8.71E-14
	*GALC*	*NDUFA4*	4.20E-03	7.34E-13
Pancreatic Cancer	*ACADVL*	*PIP5K1B*	9.27E-03	6.81E-07
	*ACADVL*	*FABP1*	5.90E-05	7.63E-06
Skin Cancer	*NOS3*	*ALAD*	1.09E-02	6.97E-19
	*PIK3C2G*	*ST3GAL1*	2.54E-03	1.47E-17

Supplementary Table S3 presents the number of shared predicted SL pairs in each SL prediction method and the pairs identified through CRISPR analysis. Notably, lung, ovarian, skin, and Leukemia cancers demonstrate the highest number of concordant SL pairs identified through the SL-scan pipeline. This finding emphasizes the importance of incorporating mutation data in metabolic modeling and the exhaustive search process.

Supplementary Tables S4-S7 provide the hypergeometric p-values for the identified SL pairs by FastSL, MCS, and gMCS, and ngMCS demonstrating their alignment with the significant outcomes obtained from CRISPR analysis. Additionally, Table 2 displays the hypergeometric results of the predicted SL pairs within the gene list of the GSMNs constructed for the SL-Scan pipeline. The table also highlights the number of significant overlaps observed between the predicted SL pairs and the results obtained from the CRISPR analysis. Additionally, we compiled the number of driver genes associated to each KO gene across diverse cancer types. This information can be used to identify KO genes that could be targeted across a wide range of scenarios (Supplementary Table S8).

**Table 2:** Results of the hypergeometric test for SL-scan and CRISPR concordant SL pairs.

Cancer	Enrichment P-value	#SL pairs(SL-scan)	#SL pairs(CRISPR)	#overlap	#total
Bile Duct Cancer	7.88E-01	580	170	5	15,066
Brain Cancer	2.43E-01	1812	865	42	41,850
Breast Cancer	9.41E-01	1313	506	18	26,784
Esophageal Cancer	9.25E-01	588	192	6	11,718
Gastric Cancer	4.89E-01	2479	613	28	55,242
Head and Neck Cancer	8.67E-01	665	223	7	15,066
Kidney Cancer	4.76E-01	285	37	2	6636
Leukemia	2.87E-02	6462	2929	140	159,030
Lung Cancer	2.44E-03	8281	4407	181	249,426
Lymphoma	6.08E-01	1215	410	22	21,762
Myeloma	1.00E + 00	234	27	0	6696
Neuroblastoma	5.76E-01	193	33	2	3348
Ovarian Cancer	2.00E-02	3623	1363	78	80,352
Pancreatic Cancer	1.49E-01	604	150	9	15,066
Skin Cancer	2.48E-01	2825	1259	57	68,634

### Validation using PRISM dataset

**Figure 3:**
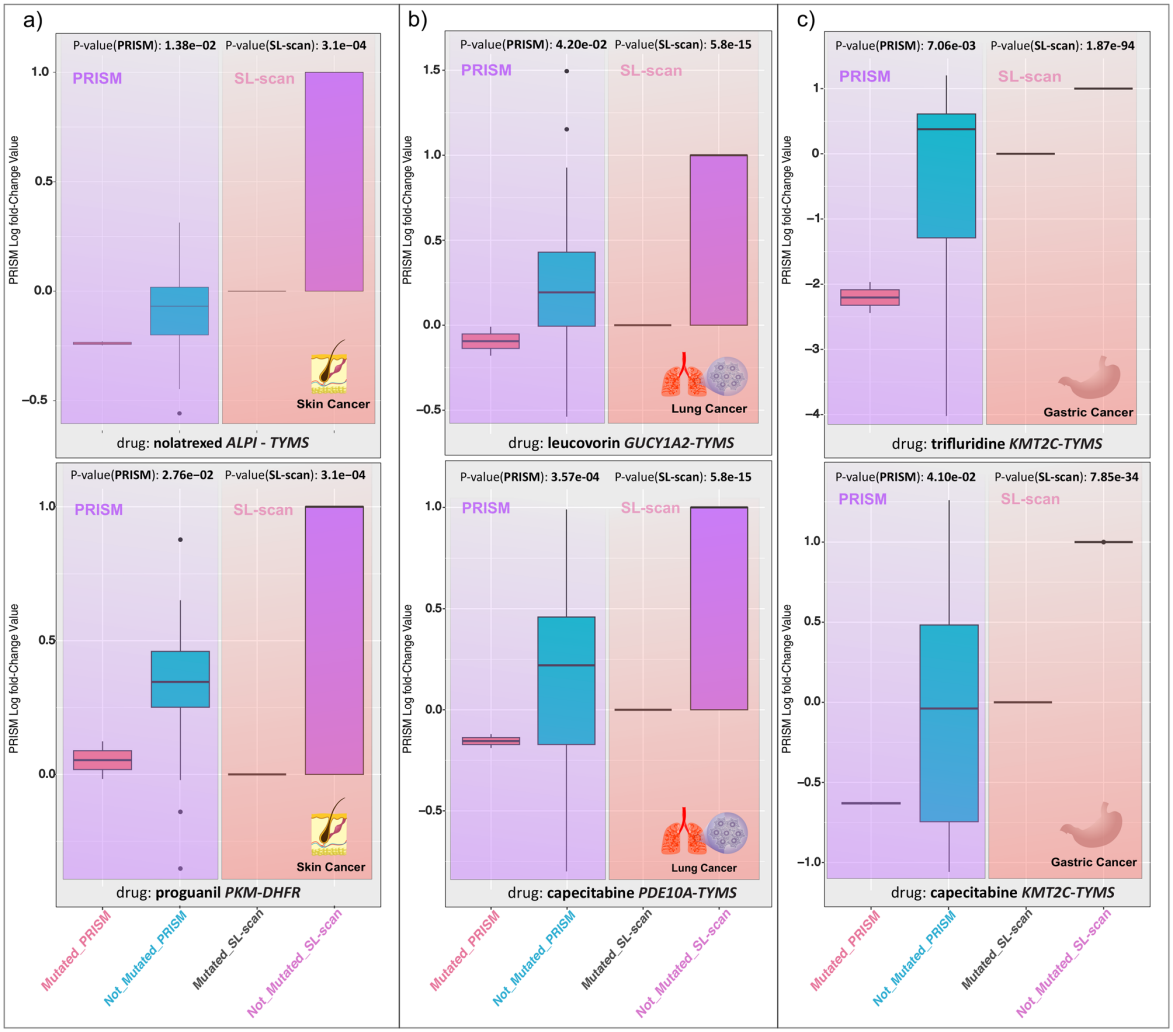
PRISM drugs for predicted SL pairs in three cancers (**a**) skin cancer *ALPI*-*TYMS* (nolatrexed) and *PKM*-*DHFR* (proguanil) SL pairs (**b**) Lung cancer *GUCY1A2*-*TYMS* (leucovorin) and *PDE10A*-*TYMS* (capecitabine) SL pairs (**c**) Gastric cancer *KMT2C*-*TYMS* (trifluridine) and *KMT2C*-*TYMS* (capecitabine) SL pairs.

To identify potential drugs for SL pairs, an exhaustive search was conducted using the PRISM dataset, similar to the CRISPR gold standard approach. No substantial drug was found among the SL pairs predicted by the FastSL, MCS, or gMCS algorithms. On the contrary, we discovered 10 different drugs for 14 different SL pairs using the SL-Scan and the PRISM dataset. Figure 3 shows six significant discovered SL pairs and the corresponding box plots for drug sensitivity between the mutated and non-mutated groups. Figure 3a shows box plots of skin cancer for SL pairs *ALPI*-*TYMS* and *PKM*-*DHFR*, treated with nolatrexed and proguanil respectively. Panel b displays lung cancer SL pairs *GUCY1A2*-*TYMS* and *PDE10A*-*TYMS*, treated with leucovorin and capecitabine. Panel c depicts gastric cancer SL pairs *KMT2C*-*TYMS* and *KMT2C*-*TYMS*, treated with trifluridine and capecitabine. Refer to Supplementary Table S9 for a comprehensive list of drugs discovered using the PRISM dataset and SL-Scan.

### Validation using shRNA dataset

Supplementary Table S10 displays the examination of the shRNA dataset using SL-scan, FastSL, gMCS, ngMCS, and MCS methods. Among these, SL-scan has successfully predicted 9 significant SL pairs that are concordant with the shRNA results. Conversely, other alternative methods have failed to predict any SL pairs consistent with shRNA outcomes across various cancer types. It is important to highlight that all other methods exhibited poor enrichment p-values as evaluated by the hypergeometric test while Sl-scan provided a better enrichment p-values.

### SL pairs partnering gene set enrichment

**Figure 4:**
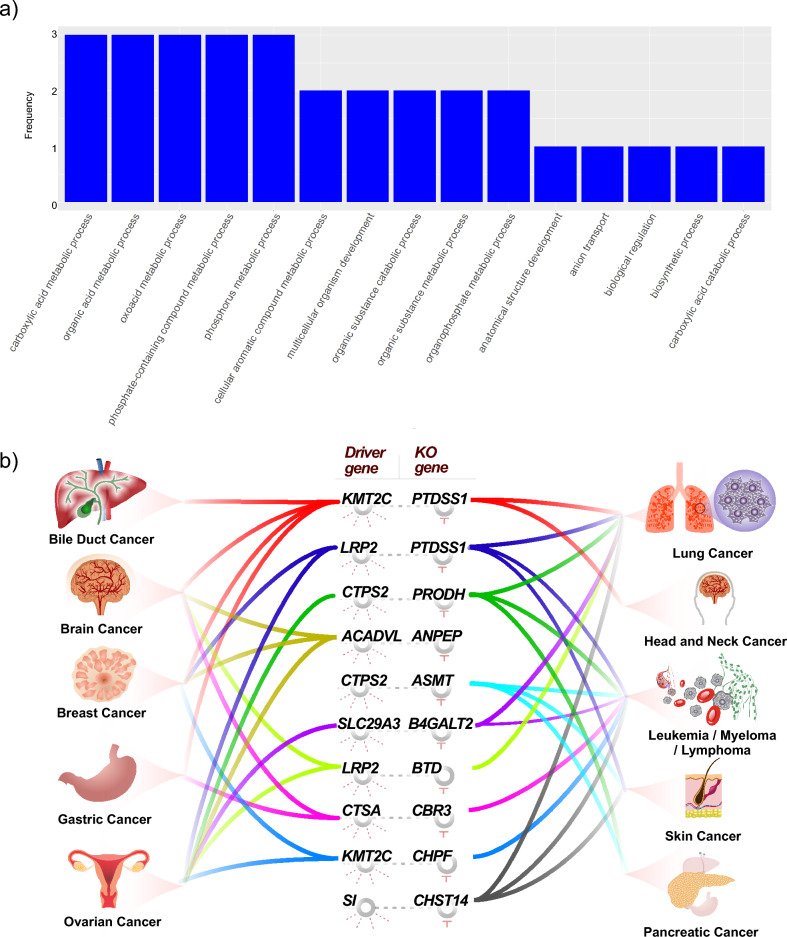
(**a**) the figure displays the top 15 most frequently enriched biological processes identified by the SL-scan pipeline, excluding general terms such as "biological process", (**b**) The distribution of SL-scan top ten concordant SL pairs among cancers.

The ranking score was determined for the partner genes of each driver gene in the predicted SL pairs using the SL-Scan approach (see "Methods" section). Figure 4a presents the top 15 most frequently enriched biological process outputs identified through the GSEA for the significant SL-scan SL pairs, excluding general terms such as "biological process". A complete version of these results can be found in supplementary material Supplementary Table S11. It is worth mentioning several significant enriched biological processes identified through the SL-scan analysis, including carboxylic acid metabolism, organic acid metabolism, oxoacid metabolism, phosphate-containing compound metabolism, cellular aromatic compound metabolism, and multicellular organism development processes. Additionally, Fig. 4b displays the top ten frequently predicted SL pairs that demonstrate concordance between the SL-scan results and CRISPR analysis across various cancer types. Notably, the *KMT2C-PTDSS1* SL pair emerges prominently in the bile duct, brain, breast, gastric, lung, and head and neck cancers, while the *LRP2-PTDSS1* SL pair is prevalent in breast, ovarian, lung, and head and neck cancers. The shared knockout gene in these recurring SL pairs may suggest its cross-cancer importance. Additionally, the driver genes *KMT2C* and *LRP2* rank among the most frequent driver genes. The recurring patterns of these SL pairs in the SL-scan outcomes, visually represented in Fig. 4b.

## Discussion

The SL-scan pipeline was developed to identify SL interactions by integrating mutation data and metabolic network modeling for different types of cancer. The pipeline involved the use of a generic human metabolic model to construct cancer-specific metabolic models, followed by exhaustive prediction of SL pairs for each cancer type. Specifically, the SL-scan pipeline begins by identifying a minimal list of reactions and translating them into corresponding genes. The pipeline then performs KO simulations for each gene in cancer metabolic models and evaluates the statistical association with mutation data.

Focusing on the common gene list between all considered approaches, the SL-scan approach successfully predicted 83 concordant SL pairs with the CRISPR analysis, significantly higher than the number of SL pairs predicted by other approaches. In particular, SL-scan exhibited the highest concordance in 13 different types of cancer. The MCS and FastSL methods also predicted one SL pair each, which concurred with the CRISPR analysis. However, the gMCS approach did not predict any SL pairs concordant with CRISPR. Additionally, we performed hypergeometric tests to assess the enrichment of CRISPR SL pairs in the SL pairs reported by SL-scan and other methods. The SL-scan method showed significant enrichment for lung cancer, ovarian cancer, and leukemia were found to be significant, while other methods did not reach statistical significance in any cancer type.

Furthermore, we discovered that KO genes linked to numerous driver genes across various cancer types play significant roles in cancer-related processes (Supplementary Table S8). For example, phosphorylation of *RRM1* regulates DNA replication and ATR inhibition vulnerability, while *RRM2* drives aggressive prostate cancer and hepatocellular carcinoma, suggesting their importance for targeted therapies ^38,39^. On the other hand, *SLC7A6* and *SLC25A19* play crucial roles in cancer by affecting amino acid transport, energy metabolism, and potential therapeutic targets^40,41^. Additionally, *PLD*, is known to be associated with lung, liver, and breast cancers ^42,43^.

The SL-scan approach has identified some promising SL pairs that were also found by the CRISPR analysis too. For example, we identified the SL pair *SLC15A2-CYP3A4* for breast cancer. Previous studies have shown that inheriting the CYP3A4*1B allele may increase susceptibility to early-onset menarche, which is a known risk factor for breast cancer ^44^. Furthermore, studies have reported that the expression levels of *SLC15A2* RNA were lower in lung cancer tissues than in normal tissues ^45^. Another example of a promising SL pair predicted by SL-scan is *PLCG1-LDHAL6A* in bile duct. The literature supports the role of the gene *LDHAL6A*, which has been found to have significantly higher expression in malignant diffuse peritoneal mesothelioma tissues compared to normal mesothelial tissues ^46^. Another example of an SL interaction predicted by the SL-scan approach is between *ACADVL* and *FABP1* in pancreatic cancer. Studies have shown that *FABP1* staining significantly increases in pancreatic adenocarcinoma samples compared to normal samples^47^.

The concordant results between the SL-scan and PRISM methods provide valuable insights from the literature. For example, we were able to predict the SL pair *KMT2C-TYMS* in gastric cancer. The analysis of the PRISM data also identified the interaction between the driver gene *KMT2C* and the drug Capecitabine, an oral prodrug of 5-fluorouracil that targets *TYMS* and is commonly used in the treatment of advanced gastric cancer ^48,49^. *KMT2C*, a frequently mutated driver gene, has been reported in the literature to have an impact on gastric cancer progression. Mutations in *KMT2C* are also associated with increased *PD-L1* positivity, indicating the existence of *PD-L1* protein that can inhibit immunological responses against cancer cells^50^. Another example is the *PKM-DHFR* SL pair predicted for skin cancer by the SL-scan approach. The driver gene *PKM* is critical in tumor metabolism and has been found to be overexpressed in various cancers, promoting tumor cell proliferation and metastasis ^51^. Furthermore, Proguanil, an anti-malaria drug that targets DHFR, has been suggested to play a role in preventing some cancers by enhancing EGFR degradation and inhibiting its downstream signaling pathway to induce autophagy ^52,53^.

Additionally, we performed a gene set enrichment analysis on partner genes for each driver gene and discovered key biological processes for each gene set, explaining each SL pair's likely biological process. Some of the most common biological processes include the carboxylic acid metabolic process, the organic acid metabolic process, and the oxoacid metabolic process, which are known as likely biological processes in cancers, and it was also reported in a study that genes differentially expressed in a mouse model of hepatocellular carcinoma were also enriched for the GO terms oxoacid, carboxylic acid, and organic acid metabolic processes^54^.

SL-scan is an approach to SL prediction that differs from other metabolic model-based methods in several ways. Firstly, instead of constructing models per cancer type, SL-scan builds models on a per-cell-line basis, enabling better modeling of heterogeneity in input expression data. Secondly, SL-scan leverages mutation data to enhance its SL predictions and gain a deeper understanding of the genetic landscape of cancer and its relationship with KO experiments. Finally, SL-scan provides statistical significance measures for predicted SL pairs, which sets it apart from deterministic approaches that solely predict SL pairs without any statistical measures.

The SL-scan approach has some limitations that need to be addressed to improve its ability to identify SL pairs and provide a better understanding of cancer dependencies. One such limitation is that it only considers metabolic genes, which may not be sufficient to capture the interactions between different types of genes involved in cancer development and progression. Therefore, it is important to incorporate other types of biological networks, such as gene regulatory networks, to identify SL pairs in a larger space of genes. Another challenge in using metabolic network modeling is the integration of gene expression data. Since gene expression data is inherently heterogeneous, it can be challenging to develop context-specific models that accurately reflect the underlying genetic state of the cell lines. Additionally, incorporating mutation data into context-specific metabolic models can also be difficult due to the lack of robust algorithms for this purpose. These challenges highlight the need for continuous refinement of computational approaches that can integrate diverse biological data sources.

To summarize, our study introduced the SL-scan pipeline, which outperformed other approaches based on metabolic network modeling. This approach has the potential to identify more promising SL interactions, opening up possibilities for the development of new and more effective metabolic therapies for cancer.

### Supplementary Information

Supplementary Information 1.

Supplementary Information 2.

Supplementary Information 3.

Supplementary Information 4.

Supplementary Information 5.

Supplementary Information 6.

Supplementary Information 7.

Supplementary Information 8.

## Data Availability

The datasets analyzed in this study include gene expression data, mutation data, CRISPR gene dependency data, and drug perturbation data from the DepMap project's cancer cell lines. These datasets are publicly available and can be downloaded from https://depmap.org/portal/download/all/. Specifically, we used CCLE_expression.csv for gene expression data, CCLE_mutations_21Q4.csv for mutation data, Achilles_gene_effect.csv for CRISPR gene dependency data, and primary-screen-replicate-collapsed-logfold-change.csv for drug perturbation data. DRIVE shRNA dataset is available here: https://data.mendeley.com/datasets/y3ds55n88r/5. Additionally, the human generic metabolic model Recon2.v04 was obtained from https://www.vmh.life/files/reconstructions/Recon/2.04. The code for the SL-scan pipeline is available at https://github.com/esnzgn/SL-scan. https://depmap.org/portal/download/all/. The gene expression data set consists of the log2 transformed transcript per million (TPM) values of 19,221 protein-coding genes from 1406 cell lines across 33 cancer types. We obtained the mutation data from the MAF file CCLE_mutations_21Q4.csv, which contains information on all somatic point mutations called in the DepMap cell lines ^27^. We used Achilles_gene_effect.csv (22Q2) for the CRISPR data set, which includes the effects of CRISPR screening on 18,018 genes in 957 cancer cell lines^3^. The drug perturbation data used in this study is the primary PRISM Repurposing dataset, which contains the results of pooled-cell line treatment with chemical perturbation for 4,686 compounds against 578 cell lines. The DRIVE shRNA dataset is available at: https://data.mendeley.com/datasets/y3ds55n88r/5. For generating cancer-tailored models, the generic input metabolic model Recon2.v04, obtained from https://www.vmh.life/files/reconstructions/Recon/2.04. The cancer-specific MutSig 2CV v3.1 TCGA MAF files were downloaded from http://firebrowse.org/.
