# Supplementary material for: SL-scan identifies synthetic lethal interactions in cancer using metabolic networks
Source: Sci Rep. 2023 Sep 22;13:15763. doi: 10.1038/s41598-023-42992-4 (PMC10516981; doi:10.1038/s41598-023-42992-4)
Supplement: Supplementary file 2 — Supplementary Information 2. [file 41598_2023_42992_MOESM2_ESM.docx]

**SL-scan Identifies Synthetic Lethal Interactions in Cancer using Metabolic Networks**

Ehsan Zangene^1^, Sayed-Amir Marashi^2*^, Hesam Montazeri^1*^

^1^ Department of Bioinformatics, Institute of Biochemistry and Biophysics, University of Tehran, Tehran, Iran

^2^ Department of Biotechnology, College of Science, University of Tehran, Tehran, Iran.

* Correspondence to Sayed-Amir Marashi [marashi@ut.ac.ir](mailto:marashi@ut.ac.ir) and Hesam Montazeri [hesam.montazeri@ut.ac.ir](mailto:hesam.montazeri@ut.ac.ir)

**Supplementary Table S1** This table provides a mapping between the cancer types as defined within the DepMap project and their corresponding counterparts in the TCGA datasets (available as a csv file in the supplementary materials, “Table S1-SL-scan MutSig mapping.csv”).

**Supplementary Table S2** presents the total predicted SL pairs using the SL-Scan, number of mutated and wild-type cell liens, t-statistics and their relevant p-value and FDR, and whether the corresponding driver gene is a significantly mutated gene (available as a csv file in the supplementary materials, “*Table S2-SL-scan predicted SL pairs and significance analysis.csv”*).

**Supplementary Table S3** presents number of predicted SL pairs and concordant SL Pairs with CRISPR results for SL-scan, FastSL, gMCS, MCS, and ngMCS methods across various cancer types. To ensure a fair comparison and evaluation of different methods, a set of universally common testable pairs was utilized consistently across all methods (available as a csv file in the supplementary materials, “*Table S3-Comparison of SL pair prediction methods with CRISPR analysis.csv”*).

**Supplementary Table S4.** Results of the hypergeometric test for FastSL and CRISPR pairs.

| Cancer | Enrichment P-value | #SL pairs  (FastSL) | #SL pairs  (CRISPR) | #overlap | #total |
| --- | --- | --- | --- | --- | --- |
| Bile Duct Cancer | 1.00E+00 | 0 | 59 | 0 | 3988 |
| Brain Cancer | 1.00E+00 | 7 | 104 | 0 | 3762 |
| Breast Cancer | 1.00E+00 | 17 | 47 | 0 | 2032 |
| Esophageal Cancer | 1.00E+00 | 1 | 43 | 0 | 2632 |
| Head and Neck Cancer | 1.00E+00 | 0 | 40 | 0 | 3212 |
| Kidney Cancer | 1.00E+00 | 0 | 15 | 0 | 2202 |
| Leukemia | 7.76E-02 | 5 | 322 | 1 | 20090 |
| Lung Cancer | 1.00E+00 | 26 | 370 | 0 | 22618 |
| Myeloma | 1.00E+00 | 0 | 3 | 0 | 970 |
| Neuroblastoma | 1.00E+00 | 0 | 9 | 0 | 1664 |
| Ovarian Cancer | 1.00E+00 | 2 | 271 | 0 | 16260 |
| Pancreatic Cancer | 1.00E+00 | 2 | 20 | 0 | 1376 |
| Skin Cancer | 1.00E+00 | 6 | 337 | 0 | 18102 |

**Supplementary Table S5.** Results of the hypergeometric test for MCS and CRISPR pairs.

| Cancer | Enrichment P-value | #SL pairs  (MCS) | #SL pairs  (CRISPR) | #overlap | #total |
| --- | --- | --- | --- | --- | --- |
| Bile Duct Cancer | 1.00E+00 | 0 | 59 | 0 | 3988 |
| Brain Cancer | 1.00E+00 | 7 | 104 | 0 | 3762 |
| Breast Cancer | 1.00E+00 | 17 | 47 | 0 | 2032 |
| Esophageal Cancer | 1.00E+00 | 1 | 43 | 0 | 2632 |
| Head and Neck Cancer | 1.00E+00 | 0 | 40 | 0 | 3212 |
| Kidney Cancer | 1.00E+00 | 0 | 15 | 0 | 2202 |
| Leukemia | 1.49E-01 | 10 | 322 | 1 | 20090 |
| Lung Cancer | 1.00E+00 | 26 | 370 | 0 | 22618 |
| Myeloma | 1.00E+00 | 0 | 3 | 0 | 970 |
| Neuroblastoma | 1.00E+00 | 0 | 9 | 0 | 1664 |
| Ovarian Cancer | 1.00E+00 | 2 | 271 | 0 | 16260 |
| Pancreatic Cancer | 1.00E+00 | 2 | 20 | 0 | 1376 |
| Skin Cancer | 1.00E+00 | 2 | 337 | 0 | 18102 |

**Supplementary Table S6.** Results of the hypergeometric test for gMCS and CRISPR pairs.

| Cancer | Enrichment P-value | #SL pairs  (gMCS) | #SL pairs  (CRISPR) | #overlap | #total |
| --- | --- | --- | --- | --- | --- |
| Brain Cancer | 1.00E+00 | 20 | 865 | 0 | 41850 |
| Breast Cancer | 1.00E+00 | 21 | 506 | 0 | 26784 |
| Esophageal Cancer | 1.00E+00 | 39 | 192 | 0 | 11718 |
| Head and Neck Cancer | 1.00E+00 | 78 | 223 | 0 | 15066 |
| Kidney Cancer | 1.00E+00 | 12 | 37 | 0 | 6636 |
| Leukemia | 1.00E+00 | 17 | 2929 | 0 | 159030 |
| Lung Cancer | 1.00E+00 | 6 | 4407 | 0 | 249426 |
| Myeloma | 1.00E+00 | 16 | 27 | 0 | 6696 |
| Neuroblastoma | 1.00E+00 | 10 | 33 | 0 | 3348 |
| Ovarian Cancer | 1.00E+00 | 11 | 1363 | 0 | 80352 |
| Pancreatic Cancer | 1.00E+00 | 12 | 150 | 0 | 15066 |
| Skin Cancer | 1.00E+00 | 10 | 1259 | 0 | 68634 |

**Supplementary Table S7.** Results of the hypergeometric test for ngMCS and CRISPR pairs.

| cancer | Enrichment P-value | #SL pairs  (ngMCS) | #SL pairs (CRISPR) | #overlap | #total |
| --- | --- | --- | --- | --- | --- |
| Bile Duct Cancer | 1 | 0 | 59 | 0 | 3988 |
| Brain Cancer | 1 | 0 | 104 | 0 | 3762 |
| Breast Cancer | 1 | 0 | 47 | 0 | 2032 |
| Esophageal Cancer | 1 | 0 | 43 | 0 | 2632 |
| Head and Neck Cancer | 1 | 0 | 40 | 0 | 3212 |
| Kidney Cancer | 1 | 0 | 15 | 0 | 2202 |
| Leukemia | 0.04731938 | 3 | 322 | 1 | 20090 |
| Lung Cancer | 1 | 10 | 370 | 0 | 22618 |
| Myeloma | 1 | 0 | 3 | 0 | 970 |
| Neuroblastoma | 1 | 0 | 9 | 0 | 1664 |
| Ovarian Cancer | 1 | 0 | 271 | 0 | 16260 |
| Pancreatic Cancer | 1 | 0 | 20 | 0 | 1376 |
| Skin Cancer | 1 | 0 | 337 | 0 | 18102 |

**Supplementary Table S8.** top KO genes in predicted SL pairs by SL-scan, along with driver genes in different cancer types (available as a csv file in the supplementary materials, *“Table S8-SL-scan KO gene frequency and associations with driver genes across cancers.csv”*).

**Supplementary Table S9.** SL-scan and PRISM concordant SL pairs and identified drugs

| Cancer | Driver gene | KO gene | Drug | PRISM P-value | SL-scan P-value | SL-scan FDR |
| --- | --- | --- | --- | --- | --- | --- |
| Gastric Cancer | KMT2C | DHFR | pralatrexate | 0.003611 | 2.10E-14 | 1.47E-11 |
| Gastric Cancer | KMT2C | DHFR | pyrimethamine | 0.0002918 | 3.66E-33 | 3.32E-30 |
| Gastric Cancer | KMT2C | TYMS | trifluridine | 0.007063 | 1.87E-94 | 3.49E-91 |
| Gastric Cancer | KMT2C | TYMS | capecitabine | 0.04097 | 7.86E-34 | 8.78E-31 |
| Gastric Cancer | KMT2C | TYMS | ftorafur | 0.03399 | 4.67E-55 | 5.80E-52 |
| Lung Cancer | GYS2 | RRM1 | triapine | 2.16E-09 | 6.76E-08 | 0.00002314 |
| Lung Cancer | PDE10A | TYMS | capecitabine | 0.0003572 | 5.86E-15 | 4.61E-12 |
| Lung Cancer | GAD2 | RRM1 | triapine | 2.16E-09 | 6.76E-08 | 0.00002314 |
| Lung Cancer | GUCY1A2 | RRM1 | hydroxyurea | 0.03236 | 6.76E-08 | 0.00002314 |
| Lung Cancer | GUCY1A2 | TYMS | leucovorin | 0.04199 | 5.86E-15 | 4.61E-12 |
| Lung Cancer | PDE1C | RRM1 | hydroxyurea | 0.03394 | 6.76E-08 | 0.00002314 |
| Lung Cancer | ENTPD6 | RRM1 | triapine | 0.004285 | 6.76E-08 | 0.00002314 |
| Skin Cancer | PKM | DHFR | proguanil | 0.02757 | 0.000310328 | 0.02237 |
| Skin Cancer | ALPI | TYMS | nolatrexed | 0.01385 | 0.000310328 | 0.02237 |

**Supplementary Table S10** presents number of predicted SL pairs and concordant SL Pairs with shRNA results for SL-scan, FastSL, gMCS, MCS, and ngMCS methods across various cancer types. To ensure a fair comparison and evaluation of different methods, a set of universally common testable pairs was utilized consistently across all methods (available as a csv file in the supplementary materials, “*Table S10-Comparison of SL pair prediction methods with shRNA analysis.csv”*)

**Supplementary Table S11** presents frequent enriched biological processes after GSEA analysis executed on significant SL-scan predicted SL pairs. General terms such as biological process are excluded from the analysis (available as a csv file in the supplementary materials, “*Table S11-SL-scan enriched biological processes.csv”*).

**Supplementary Data 1** contains FastSL, MCS, and gMCS predicted SL pairs (available as a xlsx file in the supplementary materials, “*Data 1 supplementary data1 - FastSL - MCS - gMCS SL pairs.xlsx”*).


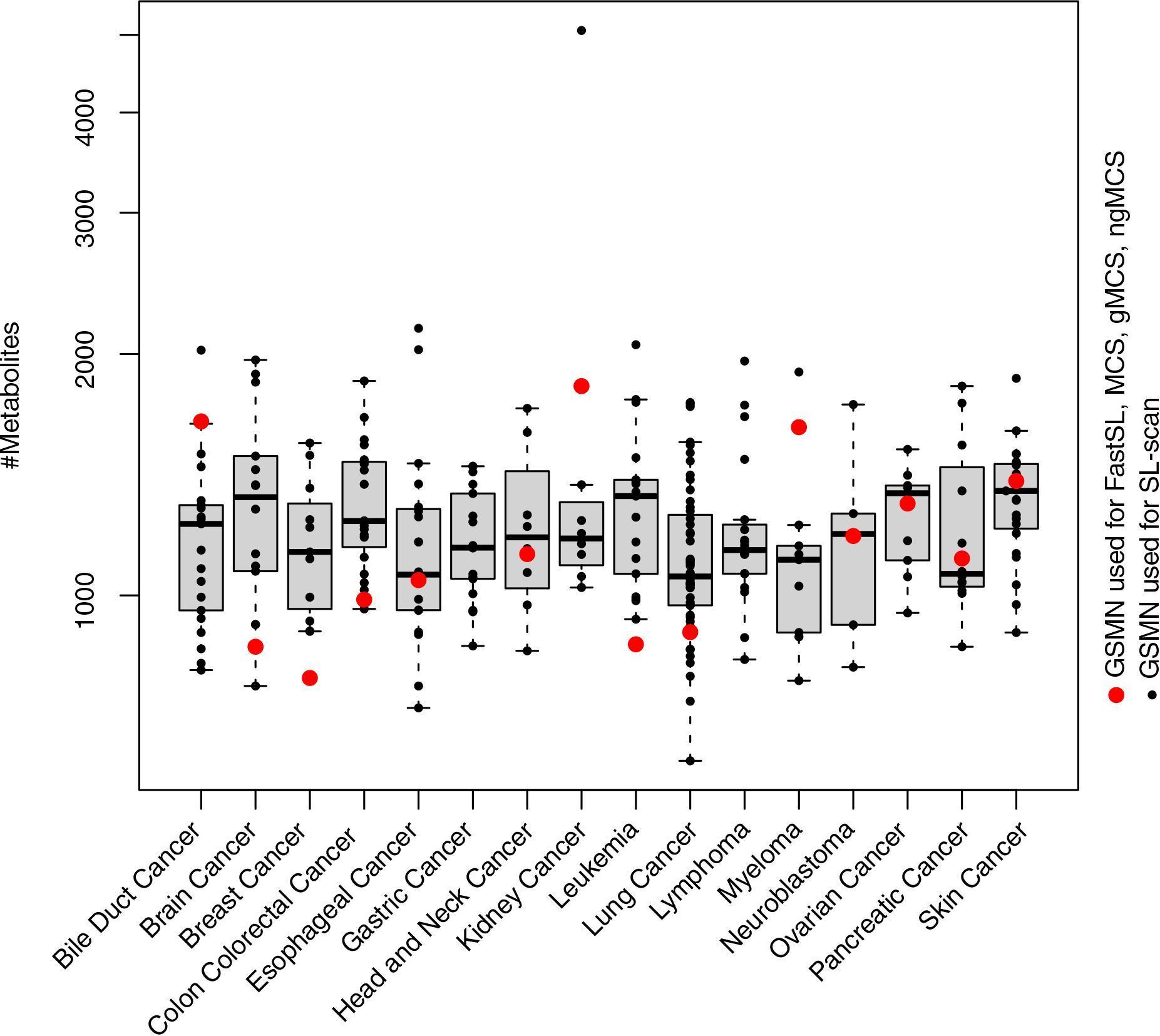


**Supplementary Figure S1** the distribution of metabolites of GSMNs in various cancer types. The corresponding values from the SL-scan pipeline are depicted as black dots, while the correspnding values for other SL prediction algorithms are indicated with red dots.


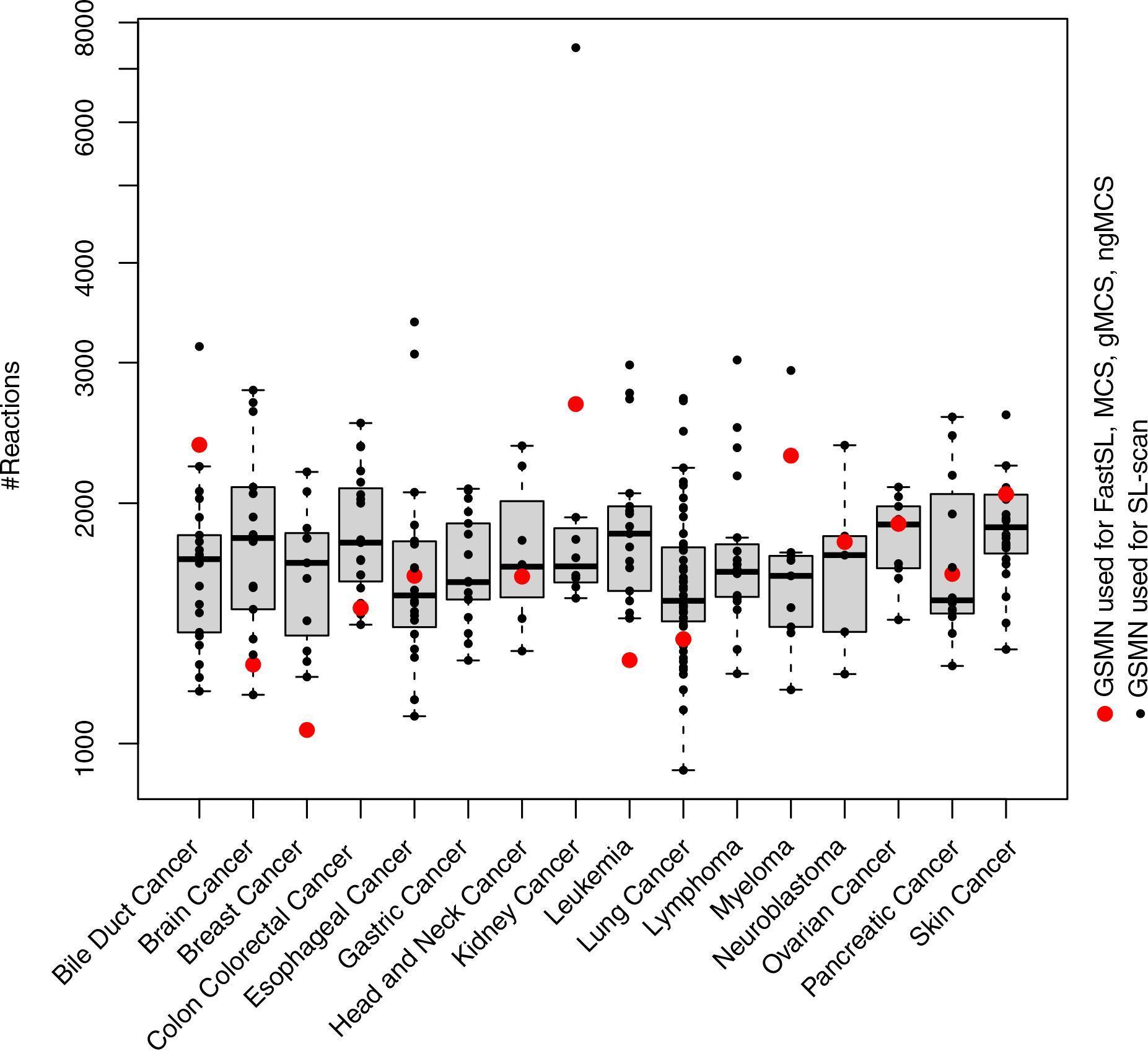


**Supplementary Figure S2** the distribution of reactions of GSMNs in various cancer types. The corresponding values from the SL-scan pipeline are depicted as black dots, while the correspnding values for other SL prediction algorithms are indicated with red dots.


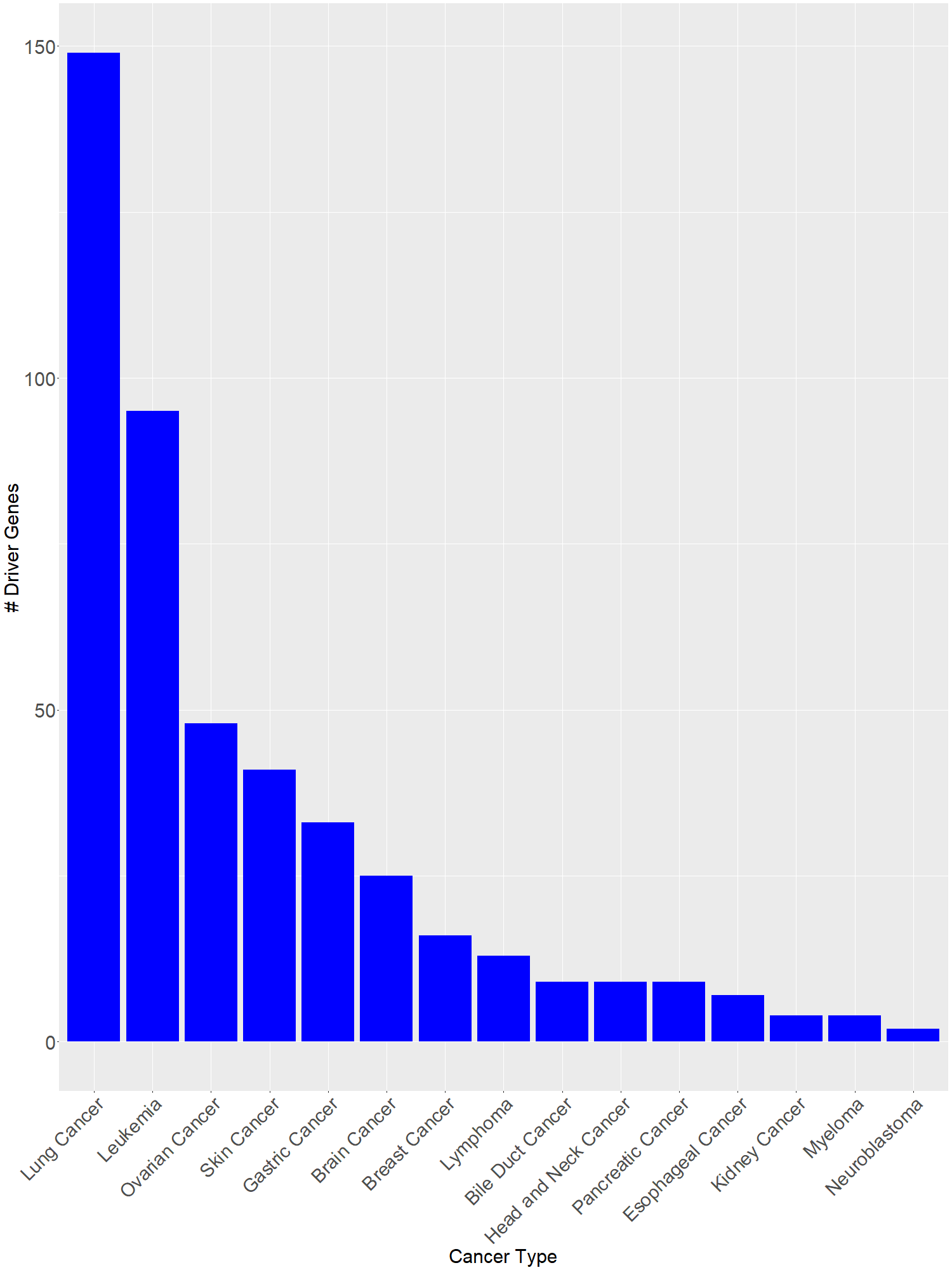


**Supplementary Figure S3** number of driver genes across different cancer types.
